# Unilateral Exophthalmos as the Initial Presentation of Acute Myeloid Leukemia in a Pediatric Patient: A Report of a Rare Case

**DOI:** 10.7759/cureus.85975

**Published:** 2025-06-13

**Authors:** Islam Erraoui, Ayyad Ghannam, Manal Azizi, Aziza Elouali, Abdeladim Babakhouya, Maria Rkain

**Affiliations:** 1 Pediatrics, University Hospital Center of Mohammed VI, Faculty of Medicine and Pharmacy, Mohammed Premier University, Oujda, MAR; 2 Pediatric Oncology, University Hospital Center of Mohammed VI, Faculty of Medicine and Pharmacy, Mohammed Premier University, Oujda, MAR; 3 Pediatric Gastroenterology, University Hospital Center of Mohammed VI, Faculty of Medicine and Pharmacy, Mohammed Premier University, Oujda, MAR

**Keywords:** acute myeloid leukemia, chemotherapy, child, diagnosis, exophthalmos, ocular manifestations, prognosis

## Abstract

Acute myeloid leukemia (AML) is a malignant hematological condition characterized by the uncontrolled proliferation of immature myeloid cells in the bone marrow, which disrupts the normal production of blood cells. Although it is a relatively rare subtype of pediatric acute leukemia, it is a significant cause of leukemia-related mortality in children. Ocular involvement in acute leukemias, though uncommon, can be a revealing sign of the disease, sometimes presenting as either an initial or secondary manifestation, especially during relapse.

We report the case of a five-year-old child presenting with progressive unilateral exophthalmos associated with fever, anemia, and chest pain, signs of general deterioration. Examinations revealed leukocytosis and circulating blasts, suggesting acute leukemia. A bone marrow aspirate confirmed the diagnosis of AML, subtype 2, with multilineage dysplasia. Orbital imaging showed exophthalmos associated with swelling of the lacrimal gland and infiltration of the extraconical fat. Chemotherapy treatment led to complete remission.

Ocular manifestations, particularly exophthalmos, are frequently associated with AML and can indicate an aggressive form of the disease or extramedullary localization. Although this presentation generally has a poor prognosis, our case demonstrated a favorable outcome, which is exceptional in the literature. This case underscores the importance of considering leukemia in the differential diagnosis of unexplained exophthalmos in children and the need for rapid diagnostic testing, including bone marrow aspiration, to confirm the disease. Early recognition and appropriate treatment are crucial for improving prognosis.

## Introduction

Acute myeloid leukemia (AML) is a malignant hematologic disorder characterized by the uncontrolled proliferation of immature myeloid cells in the bone marrow, thus impairing the normal production of blood cells. Although it accounts for about 15%-20% of pediatric acute leukemias, AML remains a particularly severe form, responsible for nearly 30% of leukemia-related deaths in children [1].

AML is rare, mainly affecting very young children or adolescents at the end of the pediatric age range. In contrast, acute lymphoblastic leukemia is the most common type of cancer in this age group, accounting for approximately 25% of oncological diagnoses before the age of 15 [2].

Among the extramedullary sites of acute leukemias, ocular involvement ranks third after meningeal and testicular localizations. It can be the initial presentation, revealing the hematologic disorder, as in the case of our patient, or it can occur secondarily during disease progression, particularly during a relapse [3].

## Case presentation

**Figure 1:**
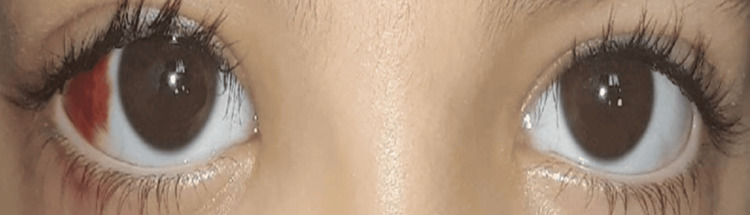
An image showing right exophthalmos, grade 1, with subconjunctival hemorrhage

The patient is a five-year-and-five-month-old child with no significant medical history, who presented with progressive right unilateral exophthalmos (Figure 1) that developed over the past three weeks. This symptomatology was associated with prolonged fever fluctuating between 39°C and 40°C for two months, right lower chest pain, intermittent watery diarrhea (four stools per day), and an anemic syndrome characterized by generalized pallor of the skin and mucous membranes and asthenia. These signs were part of a general deterioration of the patient’s condition, marked by anorexia and a weight loss of about 8 kg over two months.

Physical examination revealed growth retardation (-2 standard deviations) and right cervical lymphadenopathy measuring 1 cm, which was mobile relative to the superficial and deep planes, painless, and without local inflammatory signs. There were no signs of hepatomegaly or splenomegaly or indicators of hemorrhagic or infectious syndromes.

Ophthalmological examination revealed painless right exophthalmos, associated with moderate edema of the lower eyelid and subconjunctival hemorrhage. Visual acuity was preserved at 20/20 in both eyes, with a quiet anterior segment and a normal fundus.

Infectious tests were negative. A complete blood count and peripheral blood smear were performed, revealing leukocytosis with bicytopenia consisting of regenerative normochromic normocytic anemia and thrombocytopenia. The blood smear also showed 7% myelocytes and 45% circulating blasts, suggesting a malignant hematological disorder, probably acute leukemia. A bone marrow aspiration was performed, confirming the diagnosis of AML type M2 according to the French-American-British (FAB) classification, with multilineage dysplasia involving the granulocytic and erythroid lineages. The diagnosis was further confirmed by immunophenotyping by flow cytometry and cytogenetic analysis.

**Figure 2:**
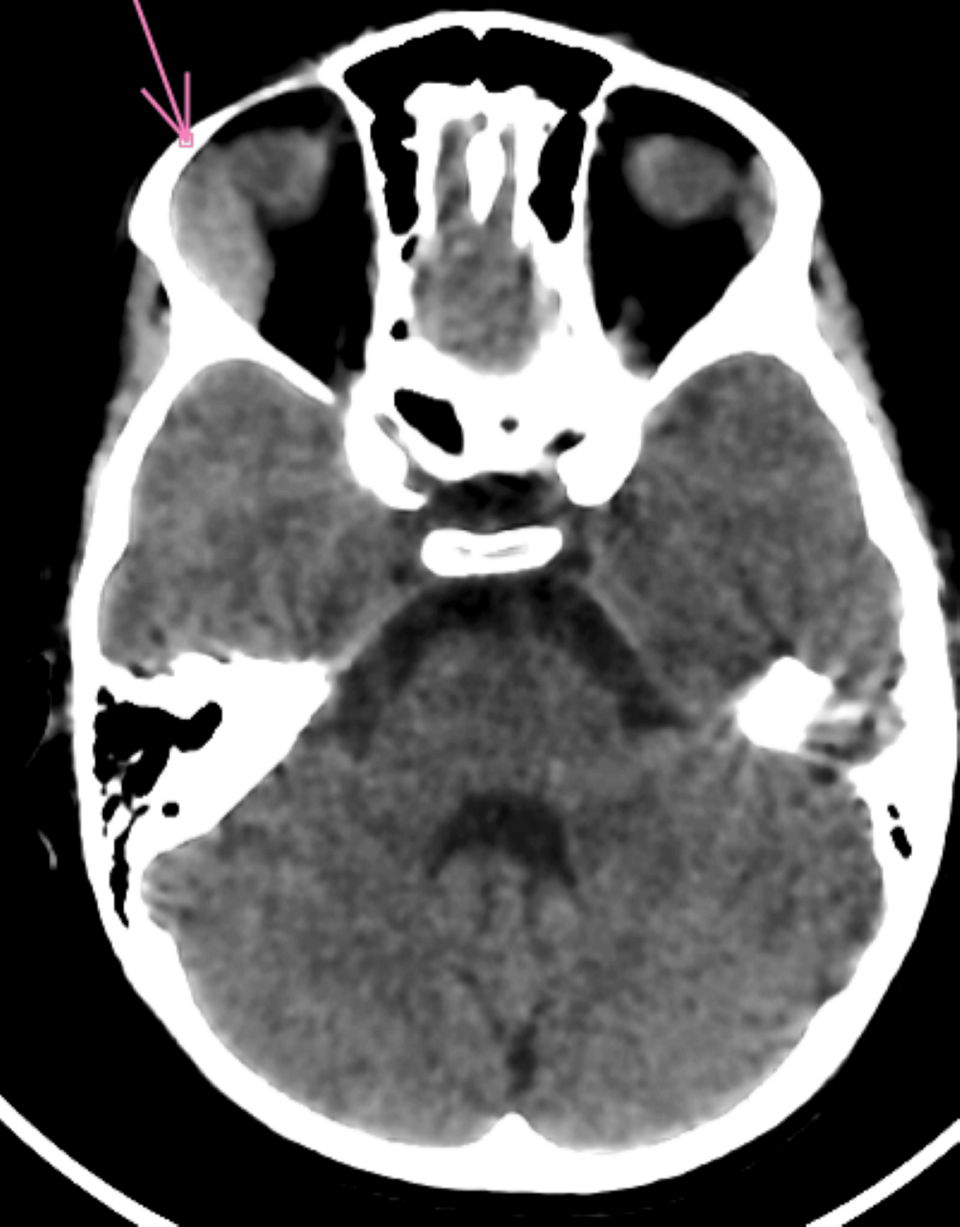
Axial non-contrast brain-orbit CT scan showing infiltration of the lacrimal gland with extraconal extension

**Figure 3:**
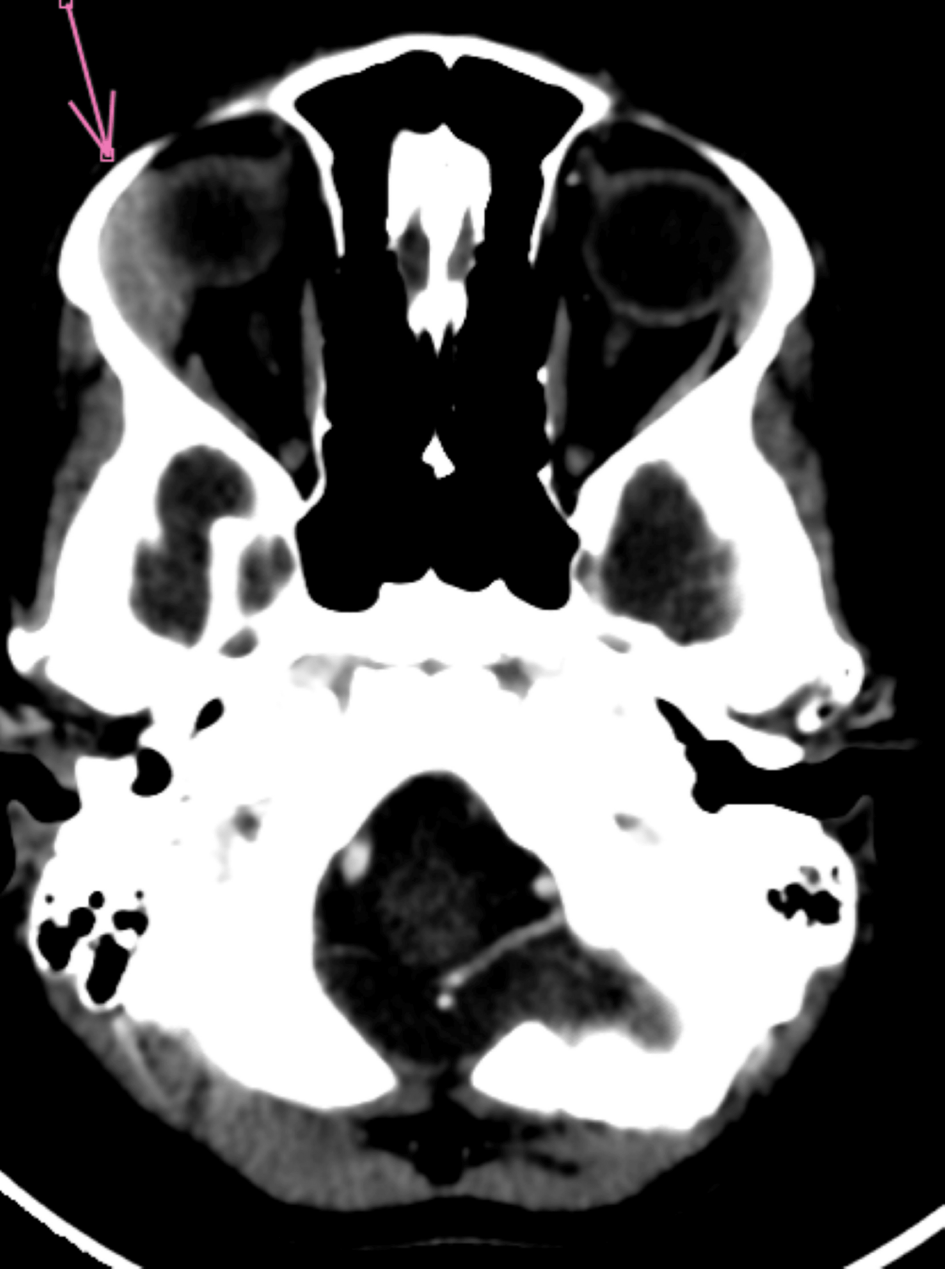
Axial contrast-enhanced brain-orbit CT scan showing infiltration of the lacrimal gland with extraconal extension

**Figure 4:**
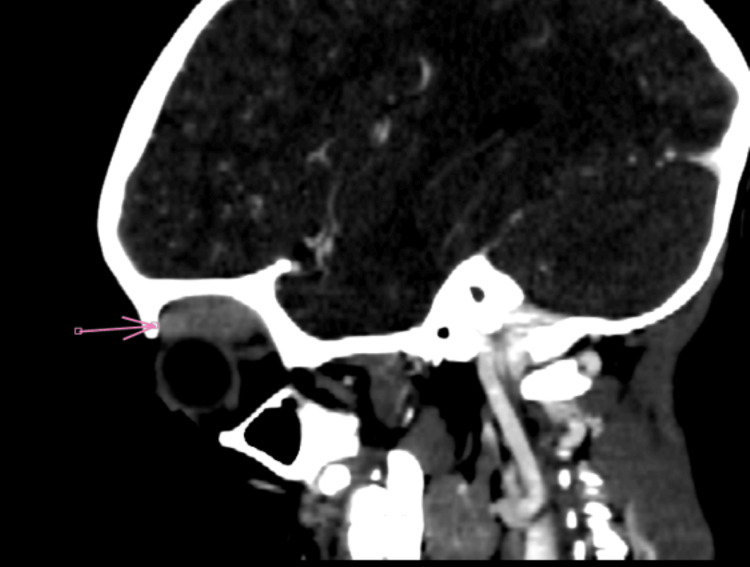
Sagittal non-contrast brain CT scan showing infiltration of the lacrimal gland with extraconal extension

**Figure 5:**
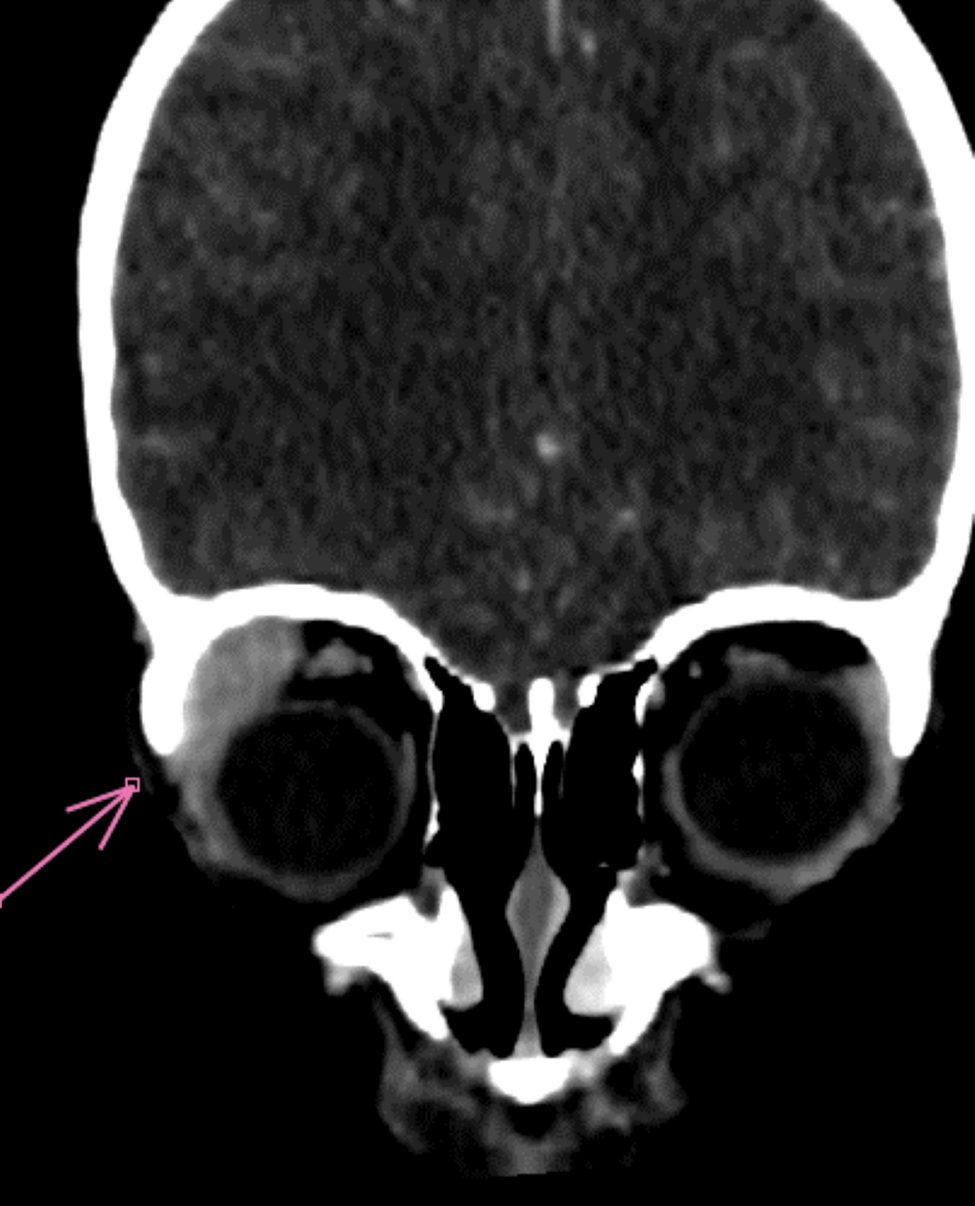
Coronal non-contrast brain CT scan showing infiltration of the lacrimal gland with extraconal extension

Orbital CT scan revealed grade 1 right exophthalmos, with swelling of the right lacrimal gland, associated with infiltration of the extraconical fat exerting a mass effect on the ipsilateral superior and medial rectus muscles (Figures 2-5).

**Figure 6:**
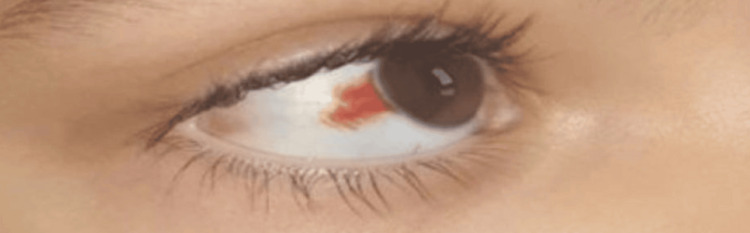
Regression of the subconjunctival hemorrhage and exophthalmos by day 8 of chemotherapy and achieved complete clinical and hematological remission following treatment according to the AML-MA 2011 protocol

The patient was treated with chemotherapy according to the AML-MA 2011 protocol, achieving an excellent clinical and hematological response, leading to complete remission of the disease (Figure 6).

## Discussion

The prevalence of ophthalmological manifestations in patients with acute leukemia varies across studies. A retrospective study conducted in the pediatric hematology department at Başkent University Hospital between 1995 and 2010 reported that 41 patients, representing 34.2% of the cohort, exhibited ophthalmic manifestations (Orhan et al. [4]). Among these patients, some presented ophthalmic signs at the initial diagnosis, while others developed them during the course of the disease.

However, much lower rates of ocular involvement have been reported in other series: 6.1% in a Moroccan cohort reported by Charif Chefchaouni et al. [5] and 15.4% in the series by Ramanarivo et al. [6]. According to data published by Gawai et al., exophthalmos was the main initial orbital manifestation, identified in approximately 43% of patients included in their study [7].

Exophthalmos is particularly noted in forms of AML with granulocytic differentiation. A case of AML2 associated with exophthalmos was described by an Algerian team in a 26-month-old child [8]. Myeloid sarcoma, an extramedullary localized tumor form of leukemia, is frequently observed in children. It predominantly affects the ocular and periocular structures, usually unilaterally, although bilateral cases have also been described. This type of sarcoma represents one of the most common orbital involvements in children in these regions [9].

In the etiological investigation of exophthalmos in children, normal results of the complete blood count and peripheral blood smear do not exclude a diagnosis of leukemia. Indeed, in AML, the migration of blasts out of the bone marrow may be delayed, resulting in normal blood tests, at least during the early stages of the disease. Therefore, a bone marrow biopsy combined with histological analysis remains essential to detect leukemic infiltration, highlighting the importance of including leukemia in the differential diagnosis of unexplained exophthalmos [4,9].

Although all forms of leukemia may potentially extend to the orbit, acute myeloblastic leukemia is the one most frequently associated with ophthalmologic signs. Several clinical studies and observations in the literature support this predominance [9]. In comparison, in acute lymphoblastic leukemia, ocular manifestations are rarer and primarily occur in specific contexts, such as medullary relapse or extension to the central nervous system. This distinction between the two leukemia entities is essential for guiding diagnosis and directing appropriate therapeutic management, particularly when faced with unexplained ophthalmologic signs in a patient at risk [10].

Orbital involvement in AML is generally seen as an unfavorable prognostic indicator. It often signifies extramedullary dissemination of the disease, which can indicate an aggressive form or herald a relapse. It is also associated with an increased risk of central nervous system involvement, further worsening the global prognosis [10,11].

Several studies have explored the potential link between ocular manifestations and vital prognosis in leukemia patients. A descriptive cross-sectional study conducted by Mirshahi et al. between 2015 and 2017 in several reference hospitals in Tehran assessed newly diagnosed leukemia patients. It notably revealed that ophthalmic involvement at the time of diagnosis was a poor prognostic factor, linked to a significant increase in mortality within the first 24 hours following disease confirmation. These data suggest that ocular involvement could reflect a severe systemic involvement or an especially high leukemic load [12]. Despite these considerations, the clinical evolution in our case was favorable, which constitutes a notable exception compared to the data typically reported in the literature. This favorable outcome may be attributed to several factors, including the young age of the patient, the relatively low tumor burden at diagnosis, and the use of the AML-MA 2011 chemotherapy regimen, known for its efficacy in inducing remission in AML.

## Conclusions

Exophthalmos, although a relatively rare clinical presentation, can be a revealing sign of acute leukemia, especially when it involves an extramedullary site. This ophthalmological manifestation, particularly when it appears suddenly and progresses rapidly, should alert clinicians to the possibility of an underlying hematological disorder. In such cases, systematic blood tests followed, if necessary, by bone marrow aspirate, constitute an essential step in the etiological diagnosis. This approach allows for the early detection of acute leukemia, especially myeloid leukemia, whose initial presentation may sometimes be misleading in the absence of obvious hematological abnormalities. The rapid recognition of this pathology and the initiation of appropriate treatment are crucial to improving prognosis, although it generally remains poor due to the severity of the systemic involvement.
